# PERCENT TIME SPENT IN AN IMPAIRED GLUCOSE STATE AND ADVERSE MUSCLE OUTCOMES: THE SWAN STUDY

**DOI:** 10.1093/geroni/igad104.2031

**Published:** 2023-12-21

**Authors:** Aleda Leis, Sidonie Kilpatrick, Elsa Strotmeyer, Carrie Karvonen-Gutierrez

**Affiliations:** University of Michigan, Ann Arbor, Michigan, United States; University College London, London, England, United Kingdom; University of Pittsburgh, Pittsburgh, Pennsylvania, United States; University of Michigan, School of Public Health, Ann Arbor, Michigan, United States

## Abstract

Sarcopenia is common among older adults, but associations of sarcopenia and diabetes are mixed. Beyond sarcopenia, relationships between glucose levels and muscle quality and power are understudied. We examined the association of time spent in an impaired glucose state and muscle outcomes. Muscle quality and power were assessed in 2015-2016 in 1,614 women (aged 66 + 2.7 years) without diabetes at baseline. Muscle quality was the average grip strength (measured in three trials using a Jamar dynamometer) per kilogram whole-body skeletal muscle mass (measured using bioelectrical-impedance analysis). Muscle power (Watts/kilogram body weight) was assessed using a 3-cycle timed stair climb: [(body weight (N)*stair height (m))/average ascent time (sec)]/body weight (kg). Fasting blood was collected at 11 visits; glucose ≥100mg/dL was considered impaired, with percent time spent in an impaired glucose state (%IGS) calculated as:(number visits with impaired glucose)/(number visits with glucose)*100 and categorized as ≤24%(n=1,203), 25-49%(n=184), 50-74%(n=136), ≥75%(n=91). Cross-sectional multivariable linear regression models were constructed for muscle outcomes with the independent predictor %IGS, adjusting for age, menopausal status, race/ethnicity, hormone use, and height. Compared to the lowest %IGS, all other groups had significantly lower mean muscle quality (β range: -0.091, -0.110 and -0.135, respectively) with the largest difference vs. highest %IGS (β= -0.135, 95%CI:-0.192,-0.077). Similar results were observed for muscle power (β range: -0.022, -0.024 and -0.020, respectively), though the highest %IGS was not significantly different. Results suggest that a longer time in an impaired glucose state from middle to older age may be associated with lower muscle function in older women.

